# TAF15 contributes to the radiation-inducible stress response in cancer

**DOI:** 10.18632/oncotarget.27663

**Published:** 2020-07-07

**Authors:** Abhay Kumar Singh, Vaishali Kapoor, Dinesh Thotala, Dennis E. Hallahan

**Affiliations:** ^1^Department of Radiation Oncology, Washington University in St. Louis, St. Louis, Missouri, USA; ^2^Siteman Cancer Center, School of Medicine, Washington University in St. Louis, St. Louis, Missouri, USA

**Keywords:** TAF15, radiation inducible, lung cancer, antibody

## Abstract

Resistance to radiation therapy is a significant problem in the treatment of non-small cell lung cancer (NSCLC). There is an unmet need to discover new molecular targets for drug development in combination with standard of care cancer therapy. We found that TAF15 was radiation-inducible using phage-displayed peptide libraries. In this study, we report that overexpression of TAF15 is correlated with worsened survival in NSCLC patients. Radiation treatment led to surface induction of TAF15 *in vitro* and *in vivo*. We genetically silenced TAF15 which led to a significant reduction in proliferation of NSCLC cells. Cells depleted of TAF15 exhibited cell cycle arrest and enhanced apoptosis through activation and accumulation of p53. In combination with radiation, TAF15 knockdown led to a significant reduction in the surviving fraction of NSCLC cell lines. To determine the importance of TAF15 surface expression, we targeted TAF15 with an antibody. In combination with radiation, the anti-TAF15 antibody led to a reduction in the surviving fraction of cancer cells. These studies show that TAF15 is a radiation-inducible molecular target that is accessible to anti-cancer antibodies and enhances cell viability in response to radiation.

## INTRODUCTION

Resistance to therapy is a significant challenge during the treatment of non-small cell lung cancer (NSCLC). NSCLC ranks among the most common type of malignancy and is the leading cause of cancer-related deaths worldwide [1, 2]. Although advancements in diagnosis and treatment have improved the survival of patients with lung cancer, the 5-year overall survival rate of NSCLC is ~19% [3]. Thus, there is an unmet need to develop novel treatment strategies for lung cancer patients. The demand is even higher for patients who have locally advanced, unresectable cancer. Targeted therapy is a growing topic of investigation for improving the current treatment strategies. The purpose of the present study is to discover additional molecular targets that complement and enhance the efficacy of standard of care chemotherapy and radiation therapy (XRT).

Our lab discovered radiation-inducible surface proteins by the use of phage-displayed peptide libraries following irradiation (IR) of *in vivo* cancers [4–6]. We identified TATA-box-binding protein-associated factor 15 (TAF15) as one protein that is expressed on the surface of NSCLC cells following irradiation. TAF15 belongs to a conserved FUS-EWS-TAF15 (FET) family of RNA binding proteins, which are key regulators of gene expression, including RNA splicing, polyadenylation, capping, modification, export, localization, translation and turnover [7, 8]. FET proteins are primarily present in the nucleus [9]; however, they also shuttle between the nucleus, cytoplasm, and the cell surface [10–12]. Thus, FET proteins have an expanded functional repertoire beyond DNA binding [13], RNA processing events like pre-mRNA splicing and mRNA transport [14], regulation [15] and interaction with a diverse number of proteins [16].

Under normal conditions, TAF15 controls cellular viability through the regulation of cell cycle and cell death-related genes [17]. Under conditions of cellular stress, stress granules, which are aggregates of protein and RNA (mostly untranslated mRNA), accumulate in the cytosol. The formation of these dense aggregates of protease-resistant complexes is needed to protect RNAs from degradation under cell stress [18]. TAF15, which possesses an RNA-binding domain, has been shown to co-localize to cytoplasmic stress granules in response to both heat and oxidative stress [19].

A previous study showed that human antibody PAT-BA4 that recognizes a variant of cell surface TAF15 inhibits cancer cell motility and cell adhesion in stomach cancer and melanoma [20]. Inhibition of TAF15 showed a growth-inhibitory effect and resulted in increased apoptosis and decreased proliferation in cancer cells [17]. In the present study, we found that IR enhanced the surface expression of TAF15 in NSCLC cell lines. We studied the effect of anti-TAF15 antibody on cells with surface associated TAF15, and its impact on cell survival when combined with IR. The results demonstrate the feasibility of targeting surface associated TAF15 as a strategy for the improvement of therapeutic efficacy in NSCLC with IR.

## RESULTS

### TAF15 is overexpressed and correlates with worsened survival in NSCLC patients

**Figure 1:**
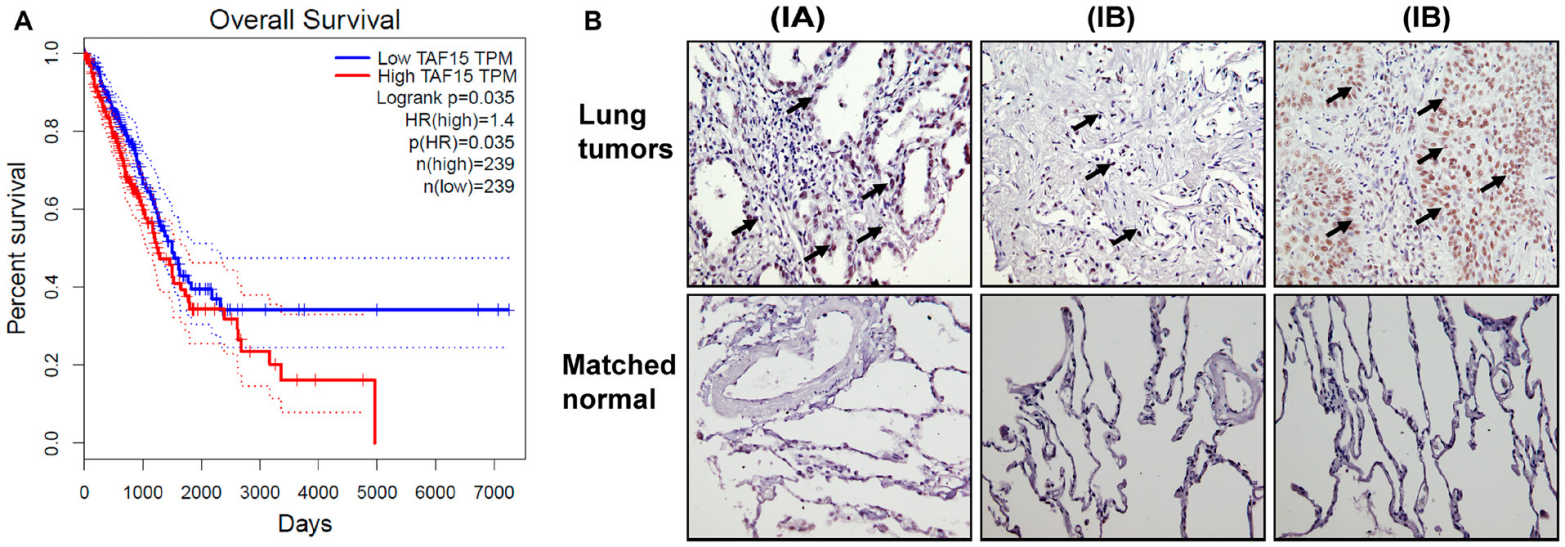
TAF15 is overexpressed in NSCLC that correlates to poor overall survival. (**A**) Kaplan Meier survival curves showing the overall survival of lung adenocarcinoma patients grouped according to their TAF15 expression levels. The survival curves were generated using the GEPIA web-browser by analyzing the TCGA RNA-Seq dataset. Patients were grouped into “High” (*n* = 239) and “Low” (*n* = 239) based on the median expression level of TAF15. High levels of TAF15 significantly correlated (*p* = 0.035, HR = 1.4) with poor overall survival of lung cancer patients. (**B**) Immunohistochemistry analysis of lung tumor tissue microarray showing expression of TAF15 in lung cancers having matched healthy tissues. The tumor tissue microarray contained cancers from 30 patients and 10 matched healthy tissue controls. Each section was represented in duplicate on the tissue array. Representative images are shown and the numbers in the parenthesis indicate the stage of cancer.

To determine if the expression of TAF15 associated with overall survival (OS) in NSCLC patients, we analyzed the RNA-Seq data for cancer (Cancer Genome Atlas (TCGA)) (3) and healthy tissue (Genotype-Tissue Expression (GTEx)) (4,5) using the web-based Gene Expression Profiling Interactive Analysis (GEPIA). Based on the median expression level of TAF15, we grouped the patients into two groups: “High” (*n* = 239) and “Low” (*n* = 239). Figure 1A shows the Kaplan–Meier survival curves representing the OS of lung adenocarcinoma patients grouped according to their TAF15 expression levels. Higher expression levels of TAF15 significantly correlated (*p* = 0.035, HR = 1.4) with a worsened OS of lung adenocarcinoma patients (Figure 1A). However, this difference in survival was not observed until 2000 days, and in the case of squamous cell carcinoma patients, we did not find a correlation between TAF15 expression levels and overall survival (Supplementary Figure 1A)

We next evaluated TAF15 expression in NSCLC patients using a tumor tissue microarray (TMA) containing NSCLC and matched healthy lung tissue (Figure 1B). The TMA contained cancers from 30 patients and 10 matched healthy tissue controls. We found high expression of TAF15 in NSCLC (black arrows, Figure 1B) and that expression levels correlated with increasing stage and grade of lung cancer. We did not find expression of TAF15 in healthy tissues (Supplementary Figure 1B).

### IR induces expression of TAF15 on the surface of cancer cells

**Figure 2:**
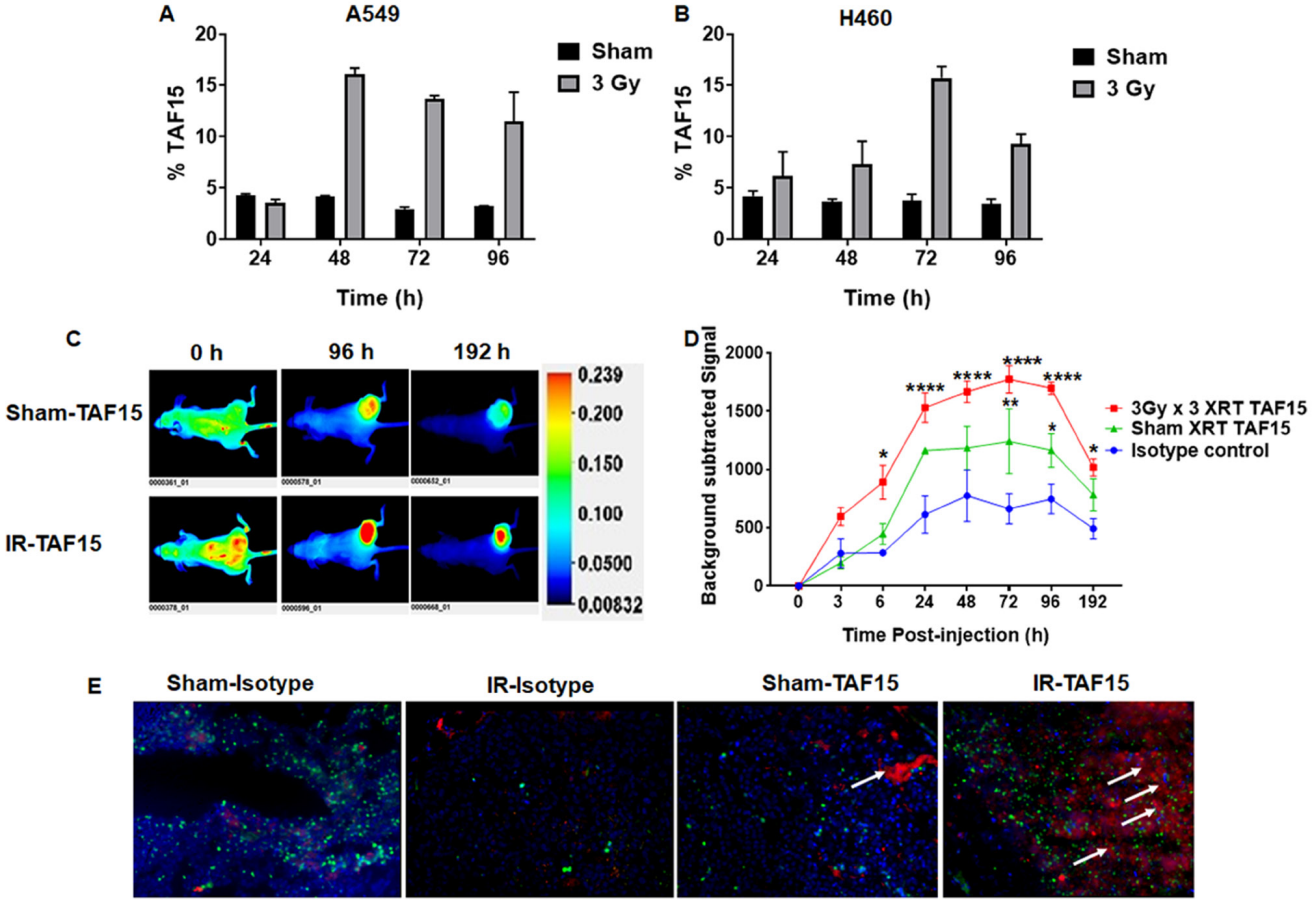
TAF15 is induced by radiation *in vitro* and *in vivo*. (**A**–**B**) Flow cytometry analysis of cell surface expression of TAF15 in A549 (A) and H460 (B) cells. NSCLC cells either were irradiated with 3Gy or sham and harvested at 24, 48, 72 and 96 h following irradiation. Cells were stained with anti-TAF15 antibody and bar diagram showing the percentage of TAF15 positive cells are plotted against the times. A two to three-fold increase in the percentage of TAF15 positive cells was observed following irradiation in both cell lines. (**C**) Representative NIR fluorescence images of nude mice after intravenous injection of IRDye 800 labeled TAF15 antibody at indicated time points. Mice were injected with A549 cells in the right hind limb and allowed to grow to 1 cm^3^ volume. Cancers were either sham irradiated or irradiated with three doses of 3Gy irradiation over a course of 24 h. Each group had three mice. Representative images are shown along with the scale bar. (**D**) Line graph showing background-subtracted fluorescence intensity of IRDye800 in sham irradiated and 3Gy × 3 irradiated A549 cancers over time compared to the isotype control. Blue lines indicate fluorescence intensity of isotype control antibody, red line and green line indicate fluorescence intensity of TAF15 antibody in 3Gy × 3 irradiated and sham irradiated cancers, respectively. Data are means ± s.e.m. (*n* = 3 mice). ^*^ *p* < 0.05, ^****^ *p* < 0.0001. (**E**) Microscopic biodistribution of the anti-TAF15 antibody in A549 cancers harvested from the hind limbs of nude mice following NIR imaging. White arrows indicate the presence of anti-TAF15 antibody (red). Nuclei were stained with DAPI and shown in blue. CD31 staining is shown in green.

We performed flow cytometry analysis to evaluate cell surface expression of TAF15 in NSCLC cells following irradiation. A549 and H460 cells were either irradiated with 3Gy or sham irradiated and harvested at 24, 48, 72 and 96 h for staining with the anti-TAF15 antibody. Supplementary Figure 2A and 2B show the overlay histograms of sham or 3Gy irradiated A549 and H460 cells, respectively. Bar graphs show that 5% of sham-irradiated cells are positive for TAF15 surface staining (Figure 2A and 2B). We found approximately a 3-fold increase in the percentage of TAF15 positive cells in A549 (at 48–72 h) (Figure 2A) and H460 (72 h) (Figure 2B) lung cancer cells following 3Gy irradiation. TAF15 surface expression remained constant in sham-irradated cells over 96 h (Figure 2A and 2B).

We also evaluated the surface expression of TAF15 in MRC5, normal lung cells. As with the cancer cells, we either irradiated with 3Gy or sham irradiated MRC5 and harvested cells at 24, 48 and 72 h for flow cytometry. We found negligible upregulation of TAF15 on the surface of MRC5 cells following irradiation. Supplementary Figure 3A shows overlay histograms of sham or 3Gy irradiated MRC5 cells. Bar graphs show that only 3% cells exhibited TAF15 surface expression following 3Gy IR at 48 and 72 h (Supplementary Figure 3B).

### IR induces TAF15 in mouse models of NSCLC

We next evaluated whether radiation induces TAF15 in subcutaneous NSCLC xenografts. We injected A549 or H460 cells into the hind limbs of nude mice to generate subcutaneous tumors. Cancers were irradiated with three fractions of 3Gy and compared to mice treated with sham irradiation. Anti-TAF15 antibody conjugated to IRDye 800 was injected via tail vein, and whole-body NIR imaging was performed (Figure 2C and 2D). Figure 2C and Supplementary Figure 3C show representative images of mice bearing A549 and H460, respectively. We found a significantly higher expression of TAF15 (*p* < 0.0001) following IR as compared to the isotype control antibody (Figure 2D) over several days. Following NIR imaging, we evaluated the microscopic biodistribution of the anti-TAF15 antibody in frozen cancer sections (Figure 2E). We found an enhanced accumulation of anti-TAF15 antibody in the irradiated tumors (white arrows) when compared to sham irradiated tumors which had little or no accumulation.

### Co-immunoprecipitation studies with anti-TAF15 antibody identify various roles of TAF15 in lung cancer

**Figure 3:**
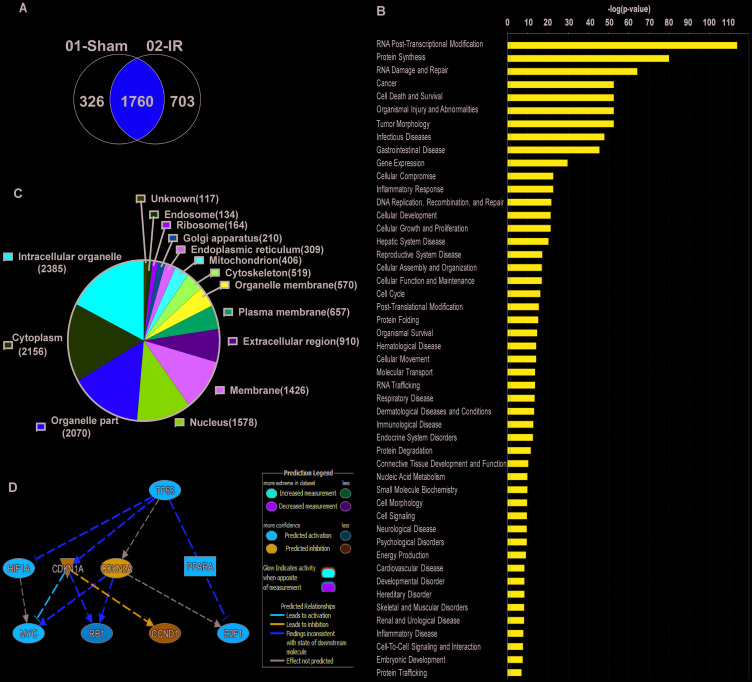
Co-immunoprecipitation studies with anti-TAF15 antibody identify various roles of TAF15 in lung cancer. A549 and H460 cells were irradiated, and TAF15 interacting partners were pulled down with an anti-TAF15 antibody. (**A**) Venn diagram showing several proteins that were pulled down in irradiated vs. non-irradiated samples. (**B**) Ingenuity pathway analysis of the pulled proteins identifies the involvement of TAF15 in various molecular functions. (**C**) Pie chart showing that TAF15 binding partners belong to various cellular components. (**D**) Predicted regulators of TAF15’s response to radiation identified by Ingenuity Pathway Analysis software.

To identify the role TAF15 plays in lung cancer cells, we performed co-immunoprecipitation of TAF15 from A549 and H460 lysates. We identified TAF15 interacting proteins using mass spectrometry. We found 326 TAF15 interacting proteins in sham irradiated lung cancer cells vs. 703 proteins in irradiated (3Gy) cells. Sham irradiated, and 3Gy irradiated samples shared 1760 interacting proteins (Figure 3A). For the identified proteins, we performed Gene Ontology (GO) analysis using the Ingenuity Pathway Analysis (IPA) software. Figure 3B shows the list of significant (-log *p*-value < 0.05) GO processes where TAF15 and its interacting partners may be involved. The most significant GO processes were RNA post-transcriptional modification, protein synthesis, RNA damage and repair, cell death and survival, cellular growth and proliferation, cell cycle (Figure 3B). We found that TAF15 and its interacting partners are localized in various cellular compartments, including membrane, nucleus, ribosomes, and organelle membranes (Figure 3C). IPA further identified possible mediators of TAF15’s response to radiation. The top predicted mediator was the TP53 (activation z-score 2.18) encoding the p53 tumor suppressor protein (Figure 3D).

### Genetic knockdown of TAF15 inhibits proliferation by arresting the cell cycle in NSCLC cell lines

**Figure 4:**
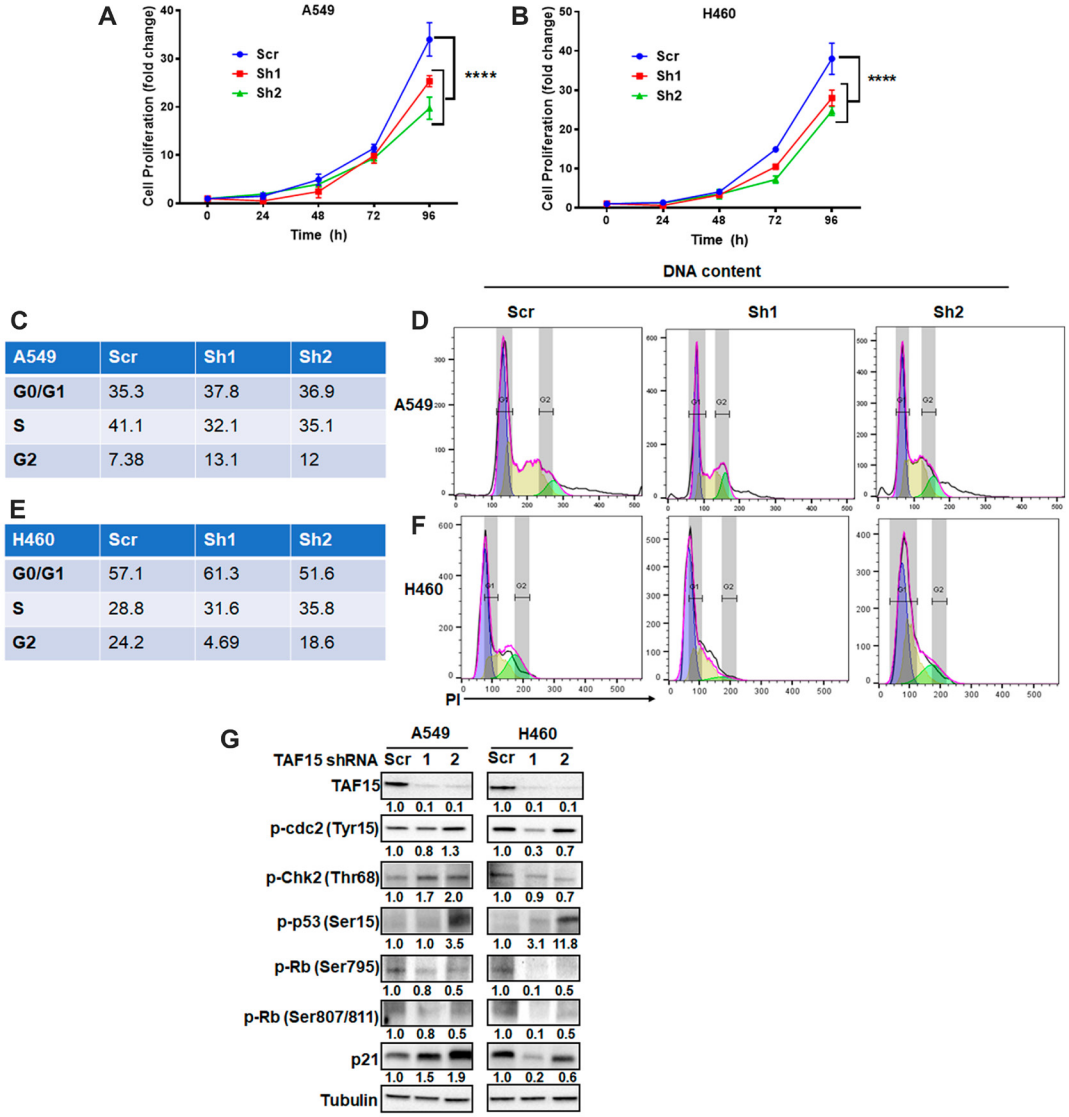
TAF15 silencing inhibits NSCLC proliferation by arresting the cell cycle. (**A**–**B**) Cell proliferation of A549 and H460 cells was monitored after the silencing of TAF15. An equal number of cells were seeded and counted at 24, 48, 72, and 96 hours. Graphs show normalized fold change vs. time in hours. ^****^ *P* < 0.001. Data represent three independent experiments. C-F Cell cycle analysis following the silencing of TAF15 in A549 (**C** and **D**) and H460 (**E** and **F**) cells. Representative histograms showing the different phases (G1: blue, S: yellow and G2: green) of cell cycle are shown in D and F and the corresponding frequencies of cells in each phase are represented in tables in C and E. (**G**) Western blot analysis for the expression of protein levels of various cell cycle regulators. Densitometric analysis was performed using the ImageJ software, and the numbers below the blots represent densities normalized to Tubulin (loading control).

To understand the role of TAF15 in NSCLC progression, we used short-hairpin RNAs (shRNAs) to knock down TAF15 in A549 and H460 cells. Two shRNAs targeting TAF15 (sh1 and sh2) reduced TAF15 protein expression by 90% when compared to scrambled shRNA (scr) as detected by western blot analysis (Figure 4G). TAF15 knockdown led to a significant reduction (*p* < 0.0001) in the proliferation of A549 and H460 cells in a time-dependent manner (Figure 4A and 4B). To evaluate whether the attenuation of proliferation following TAF15 knockdown was due to cell cycle arrest, we analyzed DNA content to determine the phases of the cell cycle (G0/G1, S, and G2). Figure 4C and 4E show the percentage of cells in each phase of the cell cycle for A549 and H460 cells, respectively. Figure 4D and Figure 4F represent the histograms showing the distribution of cells in different phases of the cell cycle following TAF15 knockdown for A549 and H460 cells, respectively. We observed arrest in the G2/M and S phase of the cell cycle. In A549, we found an increase from 7.38% (scrambled control) to 13.1% (sh1) and 12% (sh2) in the G2/M phase (*p* < 0.005) (Figure 4C). For H460, cells in S phase increased from 28.8% (scrambled control) to 31.6% (sh1) and 35.8% (sh2) (*p* < 0.005) (Figure 4E).

At the molecular level, we found enhanced phosphorylation of cyclin-dependent kinase cdc2 at Tyrosine15 residue following TAF15 knockdown (Figure 4G). TAF15 knockdown also led to the upregulation of phospho-Chk2 in A549 cells, while little change was observed in H460 cells (Figure 4G). We found the upregulation of phosphorylated levels of p53 cancer suppressor in both A549 and H460 cells following TAF15 knockdown (Figure 4G). We evaluated the Serine795 and Serine807/811 phosphorylation of the retinoblastoma protein (Rb) proteins and found a reduction in phosphorylation at these residues in both A549 and H460 cells following TAF15 knockdown (Figure 4G). Furthermore, we observed the upregulation of the p21 protein after TAF15 knockdown (Figure 4G).

### Genetic knockdown of TAF15 induces apoptosis in NSCLC cell lines

**Figure 5:**
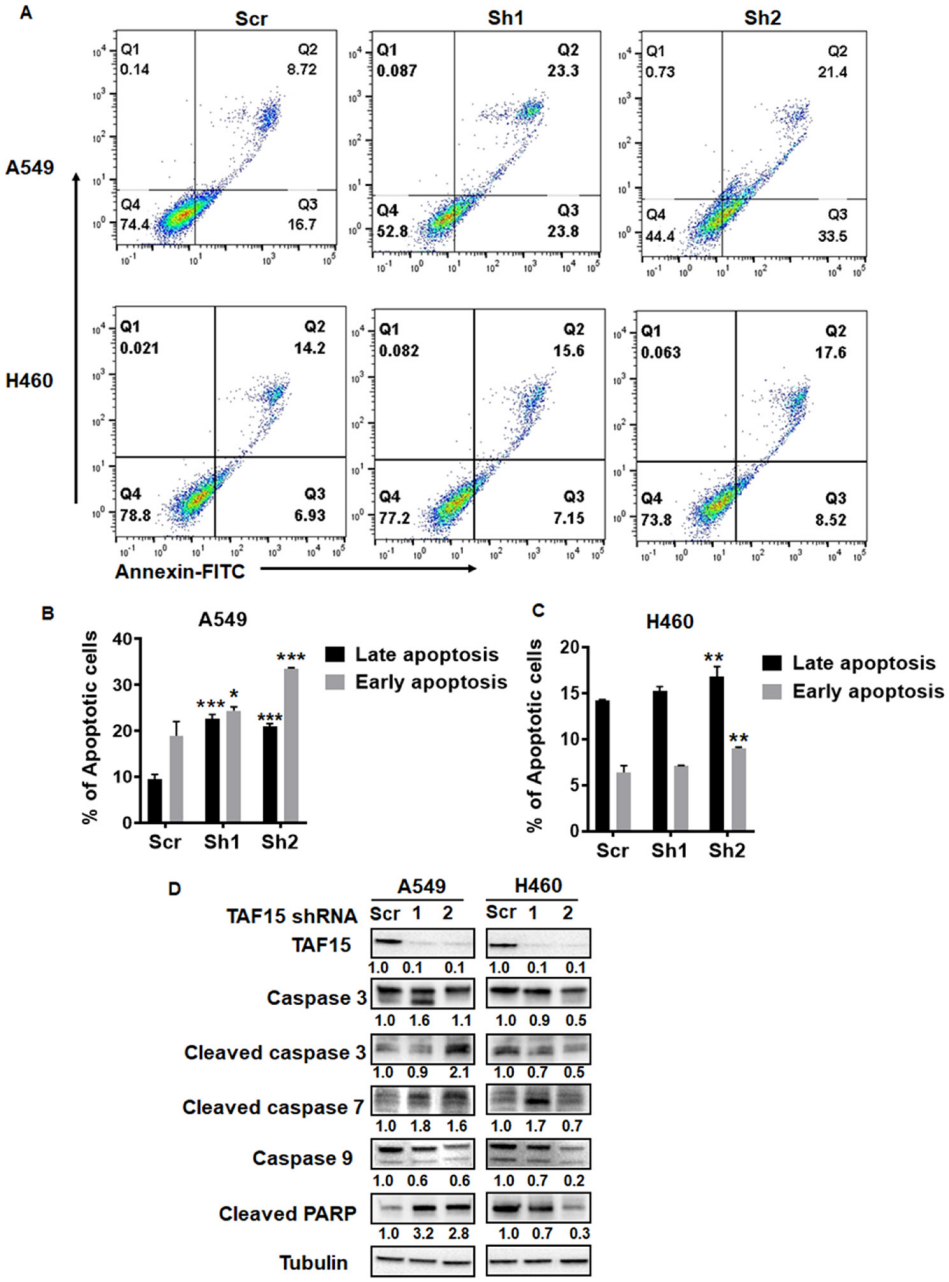
TAF15 silencing induces apoptosis in A549 and H460 cells. (**A**) Annexin V-PI staining was performed to detect apoptosis after TAF15 knockdown. Dot plots showing annexin-FITC on the x-axis and PI on the y-axis are shown. FITC and PI-positive cells (Q2, double-positive) represent late apoptosis, and FITC positive (Q3, single positive) cells represent early apoptosis. (**B** and **C**) Bar diagrams showing late apoptosis (black bars) and early apoptosis (grey bars) in A549 (B) and H460 (C) cells. Mean and SD is from three independent experiments. ^*^ *p* < 0.05, ^**^ *p* < 0.01, ^****^ *p* < 0.0001. (**D**) Western blot analysis for the expression of proteins involved in the apoptotic pathways. Densitometry analysis was performed using the ImageJ software and the numbers below the blots represent densities normalized to Tubulin (loading control).

To evaluate the role of TAF15 in cell death in A549 and H460, we measured apoptosis following TAF15 knockdown. Annexin V staining was performed to assess the percentage of cells undergoing apoptosis. Figure 5A shows representative dot plots for A549 and H460 showing Annexin V single positive (early apoptosis) and Annexin V- propidium iodide (PI) double-positive (late apoptosis) cells. TAF15 knockdown with both shRNAs led to a significant increase (*p* < 0.001) in the percentage of cells undergoing early and late stages apoptosis in A549 (Figure 5B). In H460 cells, shRNA2 led to a significant increase (*p* < 0.001) in apoptosis, whereas, shRNA1 did not show a significant difference compared to the scrambled control (Figure 5C).

We probed for known mediators of apoptosis using western blotting. We found enhanced cleavage of the effector caspases 3 and 7 in A549 and H460 cells following TAF15 knockdown (Figure 5D). We did not observe much change in the levels of the initiator caspase 9. We found the accumulation of the cleaved fragment of PARP in A549 cells depleted of TAF15 (Figure 5D). These studies further confirm that TAF15 protects A549 and H460 from apoptosis.

### Antibody targeting TAF15 enhances cytotoxicity in NSCLC cell lines

**Figure 6:**
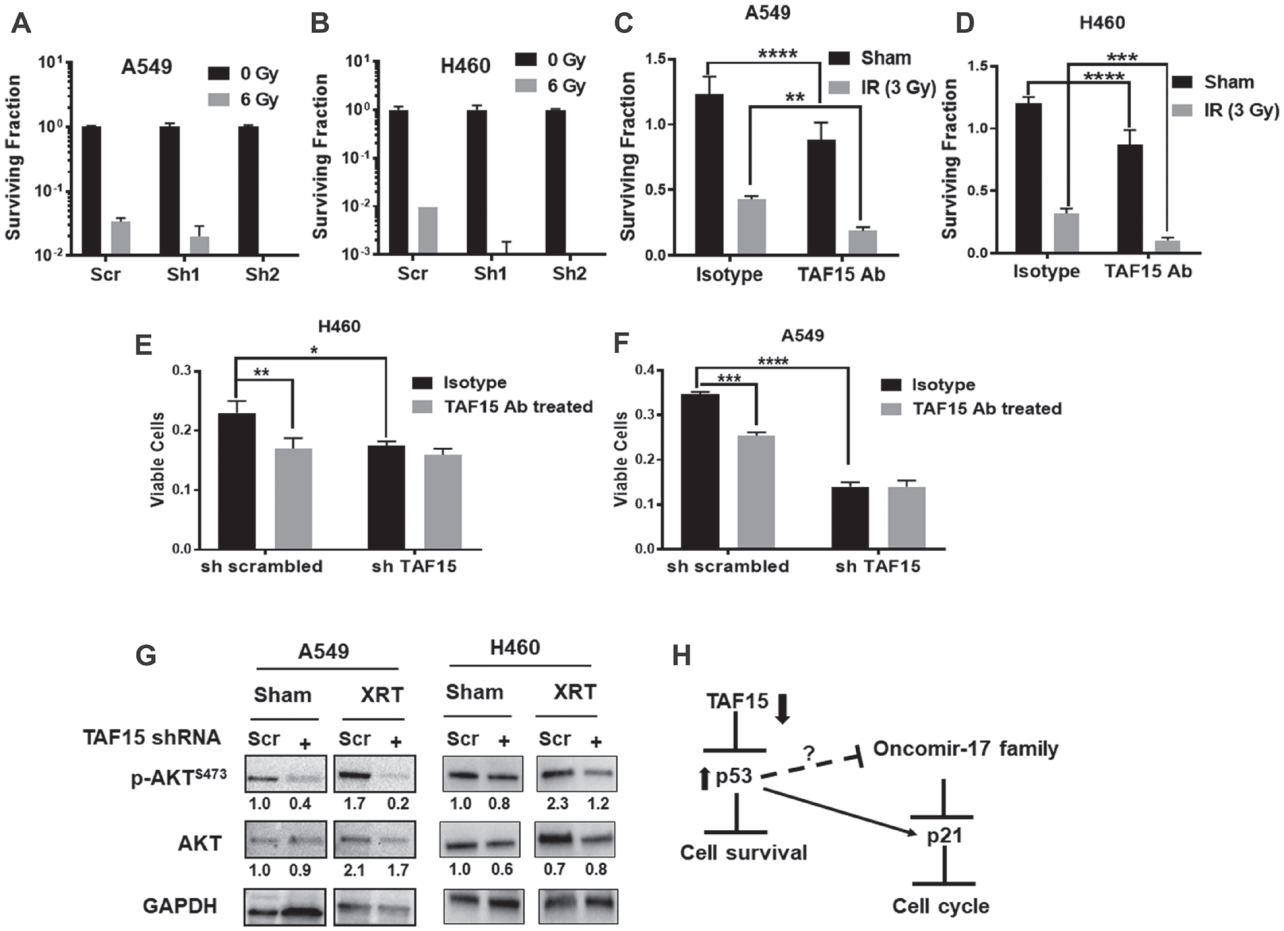
TAF15 silencing and anti-TAF15 antibody treatment sensitize NSCLC cells to radiation. (**A**–**B**) Colony formation assay performed on TAF15 knockdown cells. Representative bar grpahs at 6Gy showing log10 surviving fraction are plotted. The silencing of TAF15 sensitizes A549 cells (A) and H460 cells (B) to radiation. C-D Anti-TAF15 antibody treatment inhibits the proliferation of A549 (**C**) and H460 (**D**) cells and sensitizes them to radiation. Bar diagram showing the surviving fraction of A549 and H460 after TAF15ab in combination with 3Gy radiation. (**E**–**F**) TAF15 knockdown abrogates the effect of the TAF15 antibody on cell proliferation. A549 and H460 cells having TAF15 knockdown were treated with TAF15 antibody or isotype control and cell proliferation was evaluated by trypan blue dye exclusion assay at 96 h. Shown are the mean percentages of proliferating cells relative to the isotype treated scramble control. Mean, and SD represents three independent experiments. ^*^ *p* < 0.05, ^**^ *p* < 0.01, ^****^ *p* < 0.0001. (**G**) Western blot analysis for the expression of p-AKT (Ser473) and total AKT protein levels. Densitometry analysis was performed using the ImageJ software, and the numbers below the blots represent densities normalized to GAPDH (loading control). (**H**) Proposed mechanism of TAF15’s response to radiation in NSCLC. TAF15 knockdown leads to upregulation of p53 tumor suppressor which inhibits cell survival. P53 upregulates p21 that leads to cell cycle arrest.

We hypothesized that TAF15 induction following radiation might lead to enhanced cancer cell survival. We, therefore, investigated whether TAF15 knockdown leads to enhanced radiation cytotoxicity in NSCLC cells. We performed colony formation assays with cells having knockdown of TAF15 following irradiation (0, 2, 4, 6 and 8Gy) (Supplementary Figure 3D and 3E). We found a reduction in the surviving fraction of the TAF15 knockdown cells with increasing doses of radiation (Supplementary Figure 3D and 3E). Both A549 and H460 cells with TAF15 knockdown lost the ability to form colonies at the highest dose of radiation. Representative bar graphs at 6Gy are shown in Figure 6A and 6B.

We targeted TAF15 with a blocking antibody to determine the feasibility of developing a molecular target for the treatment of cancer. Similar to the shRNA mediated knock-down, an anti-TAF15 antibody produced a significant reduction (*p* < 0.001) in the surviving fraction of A549 cells (Figure 6C) and H460 cells (Figure 6D) compared to the isotype control. Radiation added to the anti-TAF15 antibody-treated cells further reduced the surviving fraction in both cell lines as compared to the isotype control (*p* < 0.001) (Figure 6C and 6D). To evaluate the target specificity of the anti-TAF15 monoclonal antibody, we treated the cells having TAF15 knockdown with the anti-TAF15 monoclonal antibody or isotype control antibody. The anti-TAF15 antibody significantly reduced the cell viability of scrambled shRNA containing cells compared to the isotype control (Figure 6E and 6F). The anti-TAF15 antibody did not affect the cells having TAF15 knockdown, indicating the specific targeting by the ant-TAF15 antibody in NSCLC cells.

AKT is the master regulator of cell survival. We found the downregulation of phosphorylated levels of AKT following TAF15 knockdown (Figure 6G). Radiation slightly enhanced p-AKT in both cells. However, TAF15 knockdown abrogated the radiation induction of p-AKT (Figure 6G). We found little change in the total-AKT levels in any of the treatment groups. We also evaluated phosphorylated and total AKT levels in NSCLC cells following TAF15 antibody treatment. Attenuation in p-AKT was found in A549 and H460 in response to antibody treatment. Total AKT levels were unchanged in A549 cells, as compared to a slight decrease observed in H460 cells following antibody treatment (Supplementary Figure 3F).

Based on the observations above and previous studies, we propose that TAF15 plays a protective role and mediates resistance to radiation therapy by inhibiting the tumor suppressors p53 and p21 (Figure 6H).

## DISCUSSION

TAF15 belongs to the FET family of RNA and DNA binding proteins. These proteins are involved in transcriptional and post-transcriptional regulatory processes such as transcription initiation, splicing, DNA repair, and RNA maturation. The FET family of proteins is involved in carcinogenesis; however, most of these studies have focused on FUS and EWS [17, 21, 22]. Not much is known about TAF15 in lung cancer. In the TCGA RNA-Seq lung adenocarcinoma patient dataset, although a difference in survival was not observed until 2000 days, none of the patients having high TAF15 levels in their cancer survived, as compared to 30% overall survival in patients with low levels of TAF15 (Figure 1). We did not find a correlation between TAF15 expression levels and overall survival of squamous cell carcinoma patients (Supplementary Figure 1A). RNA Sequencing data for other histological subtypes (large cell and small cell carcinoma) is not available from TCGA. Immunohistochemistry analysis of lung cancer TMA revealed a higher expression of TAF15 when compared to matched healthy lung tissue (Figure 1). Thus, TAF15 protein is overexpressed in lung cancer, and this elevated expression level correlates with cancer death.

Previously, we discovered TAF15 as a radiation-inducible lung cancer surface protein by the use of phage-displayed peptide libraries [4–6]. We performed flow cytometry analysis on non-permeabilized cells to measure the enhanced surface expression after irradiation of NSCLC. We found that irradiation of A549 and H460 cells increased the percentage of TAF15 positive cells by approximately 3-fold compared to sham-irradiated cells (Figure 2A and 2B). However, irradiation of normal lung cells, MRC5, showed negligible upregulation of TAF15 on the surface (Supplementary Figure 3A and 3B). These data indicate that low dose radiation allows enhanced TAF15 surface translocation in NSCLC cells compared to normal tissue cells. Radiation-induced surface expression of TAF15 will facilitate selective targeting of cancer by therapeutic antibodies and minimizing normal lung toxicity.

Moreover, cancer-specific binding of an anti-TAF15 antibody labeled with near-infrared dye was found during whole-animal imaging. Enhanced binding of the anti-TAF15 antibody was observed in both cancer models following irradiation. This improved binding of the antibody in the irradiated cancers began at 24 h after IR and persisted beyond four days (Figure 2C and 2D). Furthermore, the microscopic biodistribution revealed that the anti-TAF15 antibody distributed throughout cancer (Figure 2E). The biodistribution of the anti-TAF15 antibody throughout cancer sections demonstrates potential bioavailability in tumor therapy.

The co-immunoprecipitation and mass spectrometry revealed that TAF15 was interacting with proteins that localized to various cellular compartments, including the nucleus, cytoplasm, cell membrane and extracellular regions (Figure 3C). TAF15 likely localizes to the cell surface either in a complex or through the interactions with proteins present on the cell surface. Ingenuity pathway analysis also identified possible regulators of TAF15 response to radiation. We found a predicted upregulation of p53 (activation z-score 2.18), leading to the downregulation of cell cycle regulators.

To study the role of TAF15 in response to radiation, we knock down the protein in both A549 and H460 cells. Silencing of TAF15 led to a significant reduction in proliferation of NSCLC cells in a time-dependent manner (Figure 4A and 4B). Similar observations were made in HeLa cells, in which TAF15 was found to be essential for proliferation and regulation of cell cycle genes through microRNAs [17].

In our study, we found that the cells were arrested in the S and G2/M phase of the cell cycle (Figure 4). In the A549 cells, cells arrested in the G2/M phase, whereas in the H460 cells, the arrest was in the S phase of cell cycle. These subtle differences may be attributed to the differences in the cellular origin and gene signatures of these two cell lines [23]. Activated Chk2 can regulate cell cycle arrest in late G1, S, and G2 phases through phosphorylation of substrates such as Cdc25A and Cdc25C [24–26]. Furthermore, activated Chk2 can regulate not only p53-mediated apoptosis but also p53-independent apoptosis [27]. Phosphorylation of Cdc25A leads to its degradation and thereby sustained inhibitory phosphorylation of Cdc2 (cyclin-dependent kinase 1, Cdk1). In our study, we found enhanced Chk2 phosphorylation and enhanced phosphorylated levels of Cdc2 following TAF15 knockdown (Figure 4G). Unexpectedly, we observed decreased phosphorylation of cdc2 in H460 cells transfected with shRNA1. Phosphorylated Cdc2 cannot activate the Cdc2/cyclin B complex, leading to an S-phase delay or G2 arrest [26]. The active hypophosphorylated forms of RB family members block entry into the S phase by inhibiting the E2F transcriptional program [28]. We found phosphorylated levels of the Rb protein downregulated following TAF15 knockdown in both A549 and H460 cells (Figure 4G). Phosphorylated p53 levels upregulated with TAF15 knockdown. However, both TAF15 shRNAs did not produce similar levels of p-p53 upregulation. We also found the upregulation of p21 following TAF15 knockdown in A549 cells (Figure 4G). On the other hand, H460 cells unexpectedly showed a decrease in p21 levels following TAF15 knockdown. p21 binds to and inhibits the activity of cyclin-CDK2, -CDK1 and –CDK4/6 and thus regulates the G1 and S phase of the cell cycle [29, 30]. p21 may have been activated via p53, which is a known activator of p21 [31, 32]. p21 downregulation has been found to induce apoptosis [33–35]. We also found that TAF15 depletion resulted in programmed cell death in A549 and H460 cells (Figure 5A–5C). Apoptosis was mediated by the cleavage of effector caspases 3 and 7, as detected by western blot analysis (Figure 5D). PARP cleavage, which is an indicator of apoptosis, was enhanced in A549 cells; however, we did not find much accumulation in H460.

We next evaluated the importance of TAF15 in response to radiation. We performed colony formation assays and found a decrease in the surviving fraction in irradiated cells that were depleted of TAF15 compared to wild-type cells (Supplementary Figure 3D and 3E and Figure 6A and 6B). We, therefore, looked at the protein levels of phosphorylated AKT which is a well-known master regulator of cell survival. p-AKT levels were downregulated with TAF15 knockdown and further depleted in combination with radiation. These studies validated that TAF15 plays a protective role and prevents cell death from radiation therapy.

TAF15 is a molecular target for drug development. In a prior study, a human IgM anti-TAF15 antibody, PAT-B4, isolated from a stomach cancer patient, targeted a variant of TAF15 expressed on the surface of cancer cells. PAT-BA4 inhibited migration and cell adhesion [20]. In our study, we targeted TAF15 with an antibody that is specific to its ligand-binding domain. We found that the antibody targeting TAF15 enhances the cytotoxicity of cancer following irradiation. (Figure 6). TAF15 on the cell surface may have a differential role from that of nucleus bound TAF15. We evaluated AKT signaling following treatment with the anti-TAF15 antibody and found a slight decrease in p-AKT and total-AKT levels in NSCLC cells. Further studies are needed to delineate the differential roles of surface associated and nucleus bound TAF15.

TAF15 is a radiation-inducible molecular target for the development of anti-cancer antibodies. Blocking TAF15 with an antibody is a feasible approach to enhance the cytotoxicity of radiation in NSCLC. This targeting approach may lead to improved outcomes in NSCLC with enhanced expression of TAF15.

## MATERIALS AND METHODS

### Cell lines, chemicals, antibodies and irradiation

Human NSCLC cell lines A549 and H460 were obtained from ATCC and the normal lung cell line MRC5 was obtained from Sigma. A549 cells were cultured in DMEM/F-12 and H460 in RPMI media containing 10% fetal bovine serum (FBS) and 1% penicillin-streptomycin. MRC5 cells were cultured in EMEM media containing 10% FBS, 2 mM glutamine, 1% non-essential amino acids and 1% penicillin-streptomycin. All cell cultures were grown in a humidified incubator at 37°C with 5% CO_2_. All the cell lines were evaluated for mycoplasma and tested negative. The cells and the mice were irradiated with an RS2000 160kV X-ray Irradiator (Rad Source Technologies, Suwanee, GA, USA). The anti-TAF15 antibody was obtained from Abcam. All other antibodies were from cell signaling technologies. For knocking down TAF15, three different short-hairpin RNA (shRNAs) were obtained from Sigma (Saint Louis, USA).

### Flowcytometry

Flow cytometry was performed as described earlier [36]. Cells were either sham irradiated or irradiated with 3Gy and harvested at 24, 48, 72 and 96 h. Monoclonal antibodies against human TAF15 and isotype control were purchased from Abcam, USA. Anti-Rabbit Alexafluor488 secondary antibody obtained from Invitrogen, USA. For analysis of surface TAF15, cells were incubated with antibodies in FACS staining buffer (PBS containing 5% vol/vol FBS and 0.1% sodium azide). The expression level was presented as a percentage, which was determined by subtracting of isotype control. Results are presented as overlay histograms and bar diagrams. Three individual experiments were performed.

### Cell proliferation assays

Cells were seeded at a density of 10,000 cells/well in 12 well plates and cultured for 24 h, 48 h, 72 h, and 96 h. The cells were then trypsinized and counted using a ViCell cell viability analyzer (Beckman Coulter). Cell proliferation was normalized as a fold-change of control. Three independent experiments having triplicates for each treatment were performed for each cell line.

### Co-immunoprecipitation and mass spectrometry

Cell lysates for immunoprecipitation were prepared using the IP lysis buffer (Thermo Scientific). Immunoprecipitation with anti-TAF15 antibody bound to beads was performed by incubating the cross-linked bead with the lysates overnight. The eluates were analyzed by LC-MS/MS using label-free quantification techniques. Normalized spectral counts were analyzed in the Scaffold Proteome Viewer software [37] and further analyzed for pathway enrichment using the Ingenuity Pathway Analysis software.

### Cell cycle analysis

The cells were pelleted and washed with phosphate-buffered saline (PBS) and fixed in cold 70% ethanol. Fixed cells were washed with cold PBS at 4°C and suspended in Staining Buffer (RNaseA (100 μg/ml, ThermoScientific) in PBS followed by incubation at 37°C for 30 min. Cells were stained with 50 μg/ml propidium iodide for 1 h. Propidium iodide fluorescence was measured using a MACSQuant Analyzer (Miltenyi Biotech), and data analysis were performed using the FlowJo software.

### Colony formation assays

Cells were treated with 10 μg/ml of anti-TAF15 antibody and allowed to incubate for 96 h. Cells were then sub-cultured in six-well plates and irradiated with the indicated doses of radiation. The shRNA treated cells were irradiated at 0, 2, 4, 6 and 8Gy. The plates were incubated for 7–10 days following irradiation, and the colonies were stained with 0.5% crystal violet. Colonies consisting of 50 or more cells were counted using a StemiVD4 dissecting microscope (Zeiss). The survival fractions were calculated after normalizing to the plating efficiency and presented as surviving fractions relative to control [38–40].

### Apoptosis assay

Apoptosis analysis was performed using the Annexin V-FITC and PI kit (BD Biosciences) as per the manufacturer’s protocol. After staining, the cells were acquired in the MACSQuant Analyzer flow cytometer (Miltenyi Biotech).

### Immunohistochemistry

Immunohistochemistry for lung cancer (Origene) and healthy tissue microarray (Biochain) was performed as described earlier [41]. Briefly, TMA sections were de-waxed in xylene and then rehydrated in a graded alcohol series. Antigen retrieval was performed by immersing the slides in Tris-EDTA buffer (10 mM, pH 9.0) for 10 min at 125°C in a pressure cooker. Endogenous peroxidase activity was blocked with 3% hydrogen peroxide in methanol for 30 min. Non-specific binding sites were blocked by 5% normal goat serum for 1 h. Slides were incubated with anti-TAF15 antibody (dilution 1:100; Abcam) overnight at 4°C in a wet chamber. Slides were washed with phosphate-buffered saline-tween 20 (0.01%) (PBST) and then incubated with anti-rabbit HRP conjugated secondary antibody (1:5000; Sigma) for 1 h at room temperature in a wet chamber. After washing with PBST, the color was developed with a solution of 0.03% diaminobenzidine for 2 min at room temperature, and the sections were counterstained with hematoxylin.

### Western blotting

Cells were lysed using M-PER mammalian protein extraction reagent (Thermo-Fisher Scientific). Protein extracts were blotted and probed using antibodies against TAF15, p-cdc2 (Tyr15), p-Chk2 (Thr68), p-Rb (Ser15 and Ser 807/811), caspase 3, cleaved caspase 3, cleaved caspase 7, caspase 9, cleaved PARP, phospho-AKT (Ser 473), total-AKT, p21, phospho-p53 (Ser 15) (Cell Signaling Technology). To evaluate protein loading, the blots were probed for GAPDH or Tubulin (Cell Signaling Technology). The blots were visualized using the ChemiDoc-MP Imaging System (Bio-Rad) and analyzed with ImageJ Software.

### *In-vivo* near-infrared imaging

All animal studies were performed per the guidelines of the IACUC and with protocols approved by the Washington University Division of Comparative Medicine. The TAF15 antibody or isotype control antibody was labeled with IRDye 800CW as per manufacturer’s instructions (Licor). Cancers were induced by injecting A549 or H460 cells in the right hind limbs of nude mice (Charles River). The cancers were irradiated with three fractions of 3Gy or 0Gy (sham) over the course of 24 h. The cancer-bearing mice were then injected with 10 μg of labeled antibodies via the tail vein. Mice were imaged using the Pearl Trilogy small animal imaging system (Licor). Fluorescence was detected using an 800 nm channel. Images were analyzed using the Image Studio software. Background subtracted signal intensity was plotted using Graph Pad Prism software.

### Statistical analysis

Statistical analyses were performed using the Student’s *t*-test and or one-way or two-way analysis of variance (ANOVA). These analyses were performed using Prism 7 (GraphPad Software, La Jolla, CA, USA), and statistical significance is indicated in each graph where appropriate.

## SUPPLEMENTARY MATERIALS


