# Supplementary material for: TAF15 contributes to the radiation-inducible stress response in cancer
Source: Oncotarget. 2020 Jul 7;11(27):2647–59. doi: 10.18632/oncotarget.27663 (PMC7343639; doi:10.18632/oncotarget.27663)
Supplement: Supplementary file 1 [file oncotarget-11-2647-s001.pdf]

TAF15 contributes to the radiation-inducible stress response in cancer

SUPPLEMENTARY MATERIALS

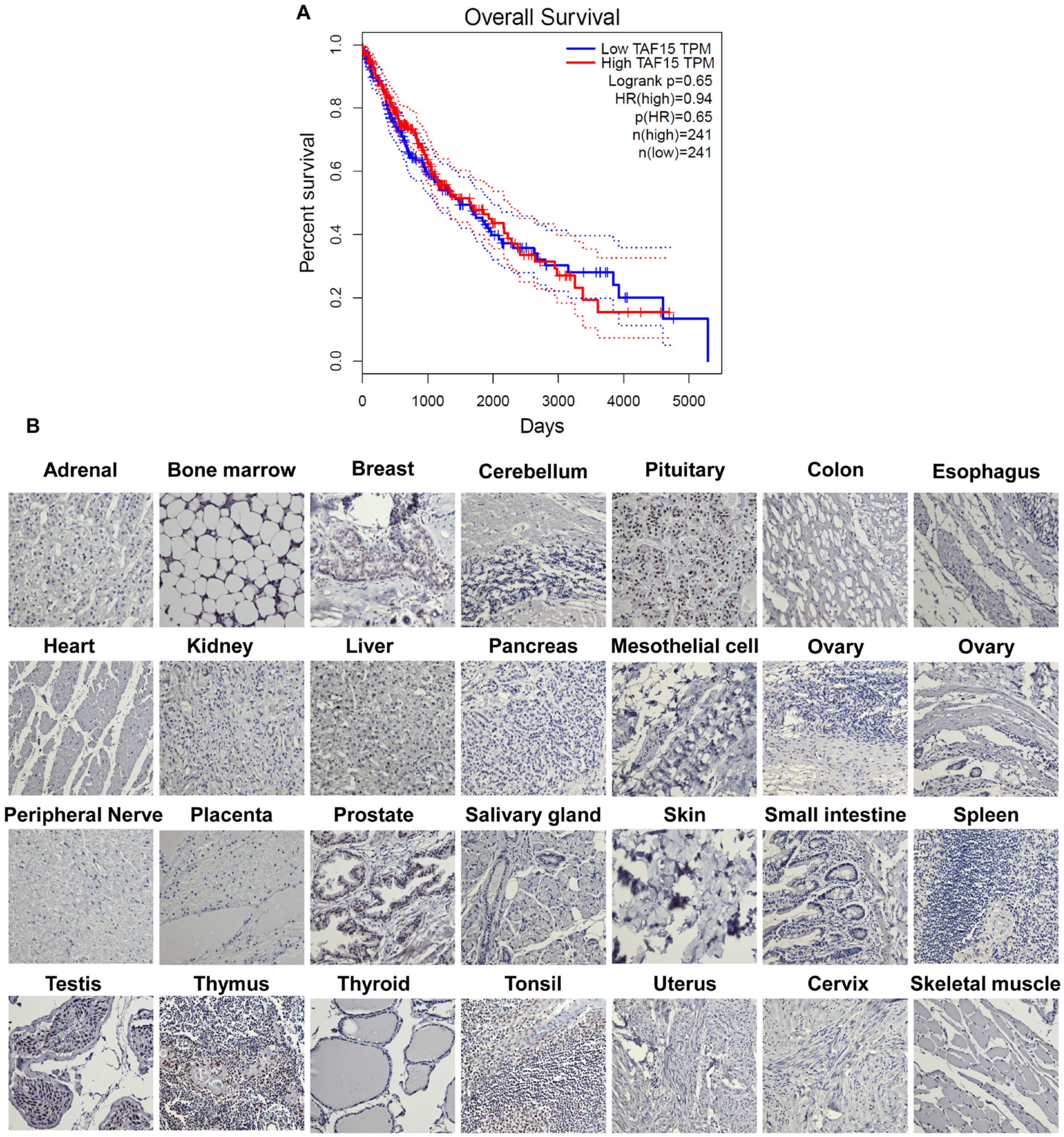

Supplementary Figure 1: (**A**) Kaplan Meier survival curves showing the overall survival of lung squamous cell carcinoma patients grouped according to their TAF15 expression levels. The survival curves were generated using the GEPIA web-browser by analyzing the TCGA RNA-Seq dataset. Patients were grouped into "High" (*n* = 241) and "Low" (*n* = 241) based on the median expression level of TAF15. (**B**) Immunohistochemistry analysis of healthy tissue microarray contained tissues from 28 different organs, and each represented in duplicate. Representative images show no expression of TAF15 in any organs of the healthy tissue microarray.

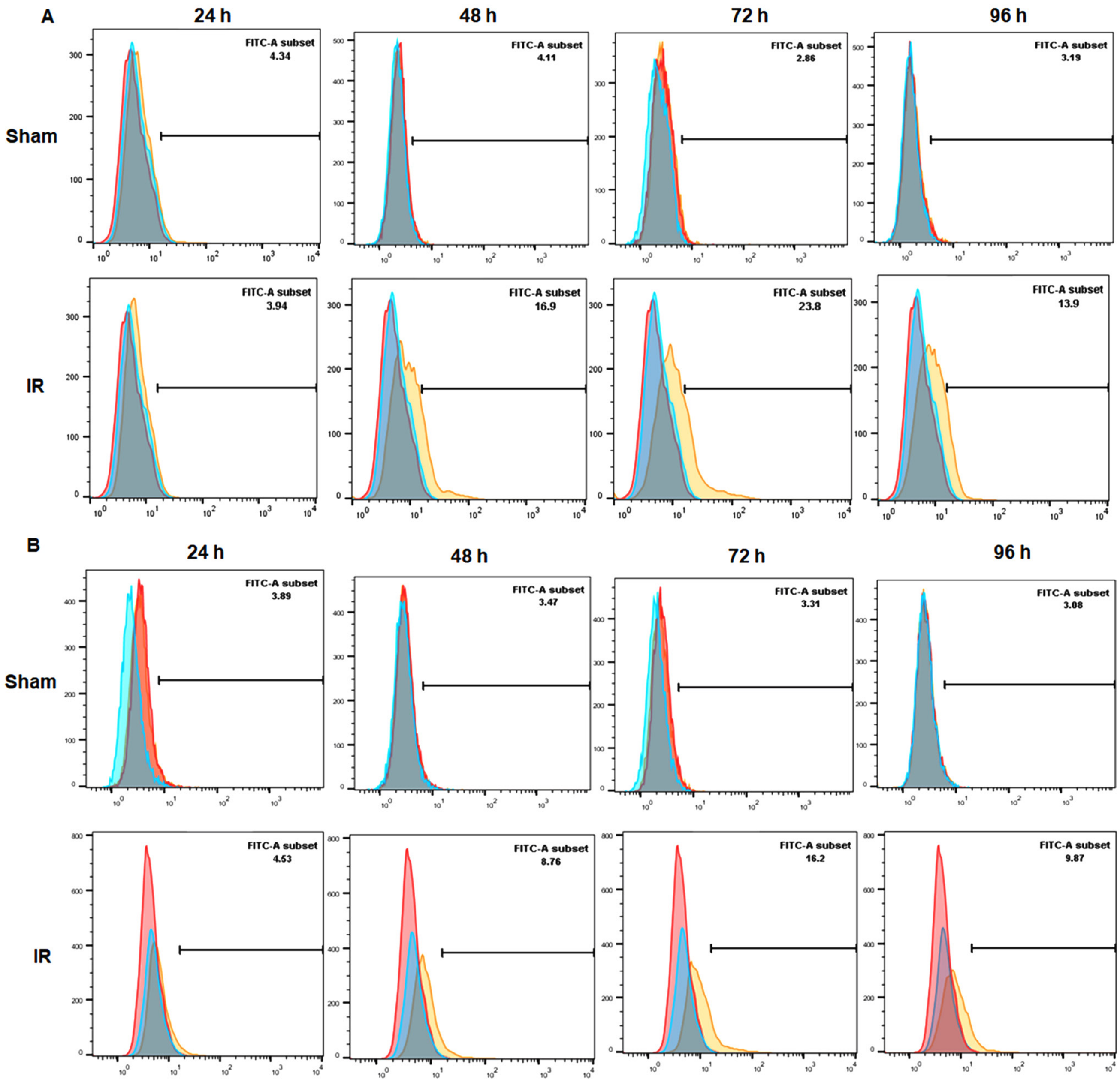

Supplementary Figure 2: Flow cytometry analysis of cell surface expression of TAF15 in NSCLC cells. (**A**) A549 and (**B**) H460. NSCLC cells were either irradiated with 3Gy or sham irradiated and harvested at 24, 48, 72 and 96 h following 3Gy. Cells were stained with anti-TAF15 antibody and representative overlay histograms (Blue: Secondary antibody control, Red: isotype control, Brown: anti-TAF15 antibody) showing staining intensity are depicted. A two to three-fold increase in the percentage of TAF15 positive cells was observed following irradiation in both cell lines.

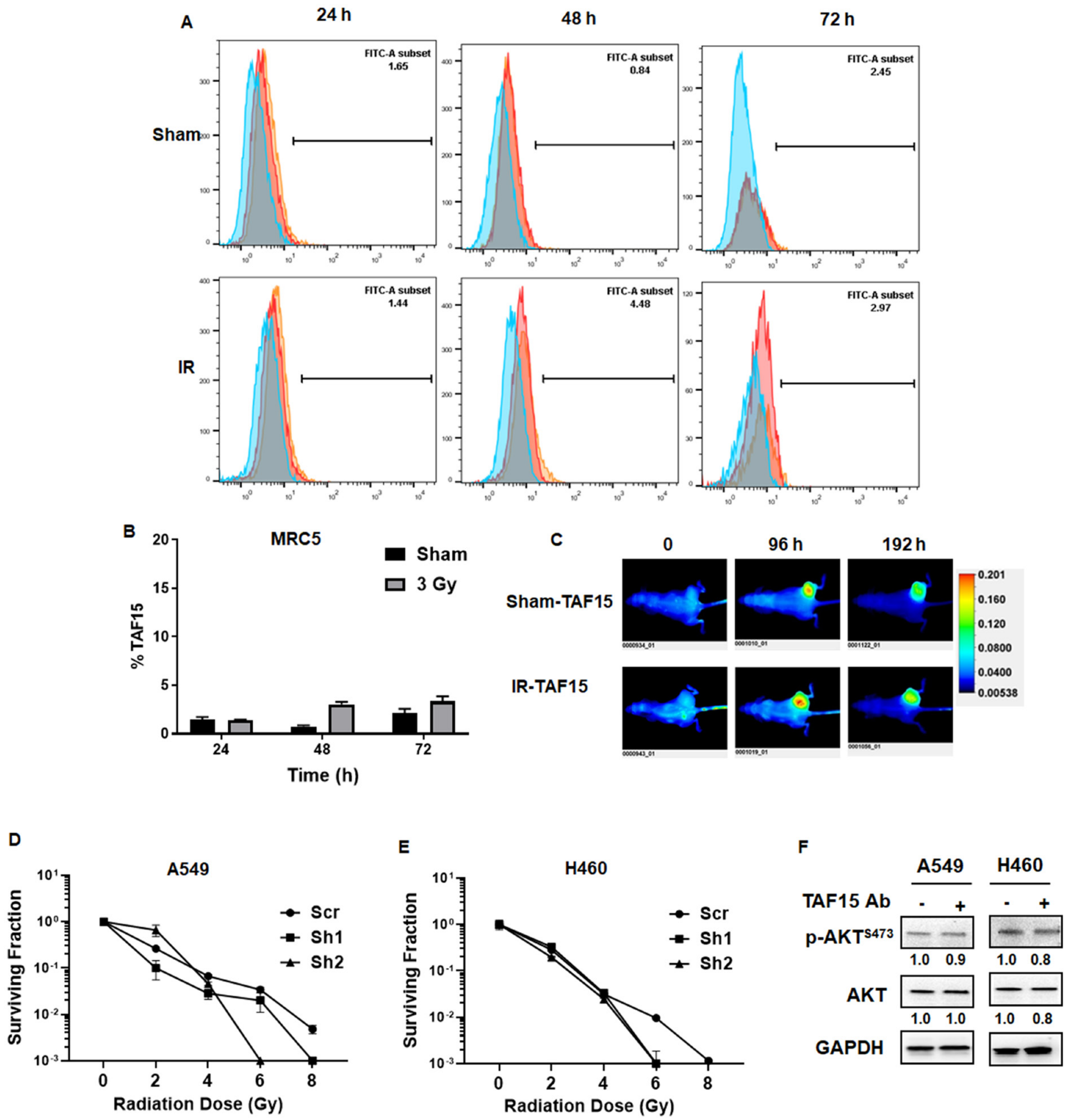

Supplementary Figure 3: (**A**) Flow cytometry analysis of cell surface expression of TAF15 in MRC5 cells. Cells were either irradiated with 3Gy or sham irradiated and harvested at 24, 48 and 72 h following 3Gy. Cells were stained with anti-TAF15 antibody and representative overlay histograms (Blue: Secondary antibody control, Red: isotype control, Brown: anti-TAF15 antibody) showing staining intensity are depicted. (**B**) Bar diagram showing the percentage of TAF15 positive sham or 3Gy irradiated MRC5 cells. (**C**) Representative NIR fluorescence images of nude mice after intravenous injection of IRDye 800 labeled TAF15 antibody at indicated time points. Mice were injected with H460 cells in the right hindlimb and allowed to grow to 1 cm^3 volume. Cancers were either sham irradiated or irradiated with three doses of 3Gy irradiation over a course of 24 h. Each group had three mice. Representative images are shown along with the scale bar. (**D**–**E**) Colony formation assay performed on TAF15 knockdown cells with increasing doses of radiations. Log10 surviving fraction plotted against the radiation doses are shown. The silencing of TAF15 sensitizes A549 cells (D) and H460 cells (E) to radiation. (**F**) Western blot analysis for the expression of p-AKT (Ser473) and total AKT protein levels following TAF15 antibody treatment. Densitometry analysis was performed using the ImageJ software, and the numbers below the blots represent densities normalized to GAPDH (loading control).
